# Water sources as reservoirs of *Vibrio cholerae*O1 and non-O1 strains in Bepanda, Douala (Cameroon): relationship between isolation and physico-chemical factors

**DOI:** 10.1186/1471-2334-14-421

**Published:** 2014-07-30

**Authors:** Jane-Francis Tatah Kihla Akoachere, Christelle Kwedjeu Pulcherie Mbuntcha

**Affiliations:** Department of Microbiology and Parasitology, Faculty of Science, University of Buea, Buea, Cameroon; Laboratory for Emerging Infectious Diseases, Faculty of Science, University of Buea, Buea, Cameroon

**Keywords:** Cholera, Water contamination, Serogoup O1, Serogroup non-1/non-O139, Physico-chemical factors, Antibiotic susceptibility

## Abstract

**Background:**

Cholera has been endemic in Douala since 1971. Most outbreaks start from Bepanda, an overcrowded neighbourhood with poor hygiene and sanitary conditions. We investigated water sources in Bepanda as reservoirs of *Vibrio cholerae*, the causative agent of cholera, determined its antibiotic susceptibility and some physico-chemical characteristics that could maintain the endemicity of this organism in Bepanda.

**Methods:**

Three hundred and eighteen water samples collected from 45 wells, 8 taps and 1 stream from February to July 2009 were analyzed for *V. cholerae* using standard methods. Isolates were characterized morphologically, biochemically and serologically. The disc diffusion technique was employed to investigate antibiotic susceptibility. Differences in prevalence of organism between seasons were analysed. Correlation strength and direction of association between physico-chemical parameters and occurrence of *V. cholerae* was analyzed using the Kendall tau_b non-parametric correlation. This was further confirmed with the forward-stepwise binary logistic regression.

**Results:**

Eighty-seven (27.4%) samples were positive for *V. cholerae.* Isolation was highest from wells. The organism was isolated in the rainy season and dry season but the frequency of isolation was significantly higher (*χ*^2^ = 7.009, df = 1, P = 0.008) in the rainy season. Of the 96 confirmed *V. cholerae* isolates, 32 (33.3%) belonged to serogroup O1 and 64 (66.6%) were serogroup non-O1/non-O139. Isolates from tap (municipal water) were non-O1/non-O139 strains. Salinity had a significant positive correlation with isolation in the dry season (+0.267, P = 0.015) and rainy season (+0.223, P = 0.028). The forward-stepwise method of binary logistic regression indicated that as pH (Wald = 11.753, df = 1), P = 0.001) increased, odds of isolation of *V. cholerae* also increased (B = 1.297, S.E = 0.378, Exp(B) = 3.657). All isolates were sensitive to ciprofloxacin and ofloxacin. Multi-drug resistance was predominant among the non-O1/non-O139 isolates.

**Conclusion:**

*V. cholerae* was found in wells and stream in both seasons. Cholera will continue to be a health threat in Bepanda if intervention measures to prevent outbreak are not implemented. Continuous monitoring of water sources in this and other cholera high-risk areas in Cameroon is necessary, for a better preparedness and control of cholera.

**Electronic supplementary material:**

The online version of this article (doi:10.1186/1471-2334-14-421) contains supplementary material, which is available to authorized users.

## Background

*Vibrio cholerae* is the causative agent of cholera, a diarrheal disease with epidemic or pandemic potential. This enteric disease is characterized by profuse watery diarrhea and vomiting, resulting in dehydration, electrolytes loss and eventually hypovolemic shock and renal failure. Without prompt medical attention, death can occur within hours [1] in 30 to 40% of cases. Cholera remains a public health concern particularly in developing countries with lack or inadequate supply of potable water, poor hygiene and rudimentary sanitary facilities. Transmission is mainly by consumption of contaminated food and water. Thus, good hygiene, appropriate sanitation and safe water are of prime importance in the fight against cholera.

*Vibrio cholerae* is classified into more than 200 serogroups based on the somatic O antigen. These are grouped into three major groups: *Vibrio cholerae* O1, *Vibrio cholerae* O139 and *Vibrio cholerae* non-O1/non-O139. Epidemic cholera is caused by *Vibrio cholerae* O1 and O139 strains. Strains of the non-O1/non-O139 serogroup have been associated with sporadic cases of diarrhea disease [2, 3]. The epidemic potential of the organism is conferred by the production of a potent cholera toxin (CT) and an adhesion factor toxin-co-regulated pilus (TCP). However, *ctxAB* and *tcp* genes which code for these major virulence determinants have also been identified in environmental strains of some non-O1/non-O139 *Vibrio cholerae* [4, 5], and their presence have been attributed to serogroup conversion through gene transfer mechanisms such as natural transformation [6] and transduction [7]. Thus for an effective diarrheal disease prevention programme in cholera endemic localities, it is important to include the surveillance for non-O1/non-O139 strains in environmental samples.

*V. cholerae* is autochthonous to the aquatic environment explaining its detection in diverse aquatic environments (brackish water, marine and fresh water) and the occurrence of sporadic outbreaks of cholera in non-endemic localities has been associated with floods contaminating water sources or imported cases. It can survive in some aquatic environments for months to years, in association with zooplankton and other aquatic organisms [8]. Both laboratory and field studies [9, 10] have shown that its occurrence in the aquatic habitat is influenced by physico-chemical characteristics. Under adverse environmental conditions, it has been shown to exist in a viable but non-culturable state which could revert to a transmissible state when climatic conditions become favourable, suggesting that in endemic regions, the aquatic environment may serve as a reservoir for this pathogen in the absence of an outbreak of cholera. Thus a deep understanding of these factors would facilitate disease prediction and implementation of timely measures for prevention of outbreaks.

Treatment of cholera involves prompt rehydration to replace lost fluids and electrolytes, and administration of antibiotics. Although rehydration prevents death, antibiotics administration has been very crucial in reducing the shedding of the pathogen, preventing disease spread, treating severely ill patients by reducing the volume of diarrhea, and consequently, reducing the duration of illness and hospitalization. Like for most enteric pathogens, there are reports of changing antibiotic susceptibility pattern of *Vibrio cholerae* isolated from cholera cases in endemic regions due to the emergence and spread of multidrug resistant strains [11]. Worrisome also, are reports of *Vibrio cholerae* strains form environmental sources bearing R plasmids transferable by intraspecific and intergeneric matings [12]. These findings show that the organism is capable of acquiring or spreading resistance factors and thus the aquatic environment could serve as a reservoir for drug-resistant strains. Therefore in cholera endemic regions, surveillance of antibiotic susceptibility patterns of the organism must include isolates from aquatic sources to identify effective agents for treatment so as to avert the unpleasant consequences of treatment failure.

Cholera has been endemic in Douala, the economic capital of Cameroon since 1971 [13] when the on-going 7^th^ pandemic hit the African continent. Outbreaks have been occurring almost every two years and have often started in Bepanda [14], a slum area with poor hygiene and sanitary conditions*.* Thus, as inhabitants of Douala are constantly threatened by outbreaks of cholera, it is important to study the reservoirs (particularly during a period with no disease outbreak) and environmental factors maintaining the endemicity of *Vibrio cholerae* in this region. This study was aimed at isolating and characterizing *V. cholerae* strains from water sources in Bepanda, determining the susceptibility of isolates to antibiotics and evaluating some physico-chemical parameters that could be supporting the survival of the organism in this locality, so as to generate information that could be exploited for cholera prevention and management.

## Methods

### Study area

Douala being the economic capital of Cameroon and major seaport handles the country’s major imports and exports and has the highest number of industries. These factors attract more people to the city resulting in rapid population growth and urbanization. The population of the city of Douala is estimated at over 3 million inhabitants. Douala’s climate is of the tropical monsoon type, with warm and humid conditions. Rainfall occurs throughout the year, with August being the wettest month and December the driest month. Access to clean and safe water or basic sanitary facilities particularly in densely populated areas is poor [13, 15]. In such areas, most people obtain water from unprotected shallow wells that are rarely disinfected [16]. The main consequence of the use of contaminated water has been the occurrence of several outbreaks of cholera [14, 17].

**Figure 1:**
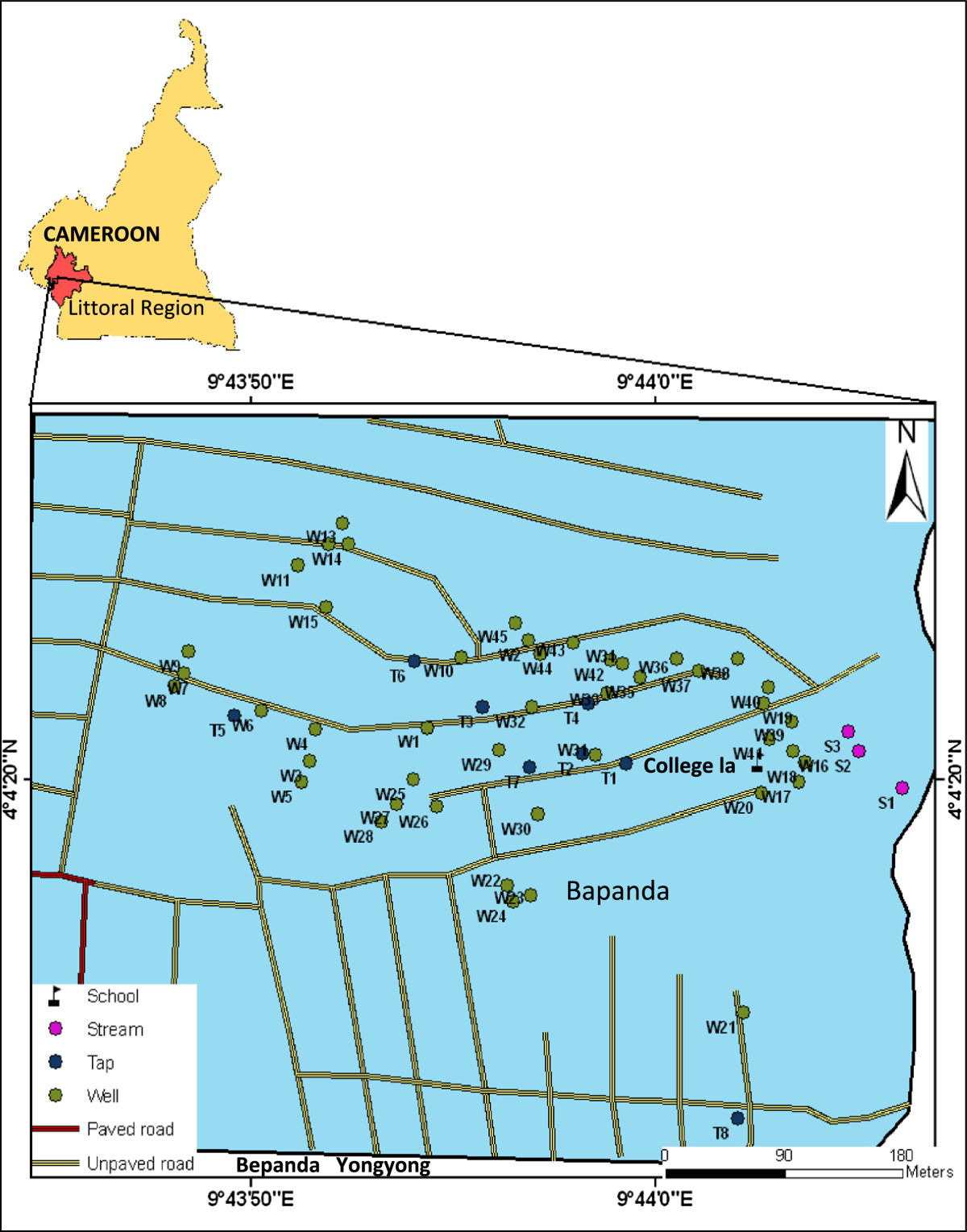
**Map of the study area indicating sampling point (Source: Douala Urban Council).**

Bepanda is one of the most densely populated neighbourhoods of Douala (Figure 1). Inhabitants of Bepanda have limited access to adequate drinking water sources and improved sanitation facilities, as well as insufficient living area. Dug-wells are the main source of water supply. The stream in this area is a major site of domestic waste disposal including leachates from nearby latrines. It also serves as a recreation site. Most cholera outbreaks have started in Bepanda [13, 14] since 1971 when the disease arrived Cameroon. Following the 2005 epidemic, this neighbourhood has had its name informally extended with cholera tag: "Bepanda cholera", to show it is renowned for cholera outbreaks.

### Study design

Water samples were collected from 3 sources (wells [n = 45], taps [n = 8], stream [n = 1]) monthly from February to July 2009 and analyzed for *V. cholerae* using standard methods. Isolates were characterized morphologically, biochemically and serologically. Susceptibility of *Vibrio cholerae* O1 and non-O1/non-O139 to antibiotics was determined. The relationship between some environmental factors (temperature, pH and salinity) and the occurrence of *Vibrio cholerae* was studied.

### Sample collection

Three hundred and eighteen (318) water samples were collected from selected wells and taps as described by Cheesbrough [18], using sterile plastic containers (500 mL). Briefly, a sterile wedge was tied with a rope (about 15 m long) to increase the weight of the container. The set up was introduced in the well and the wedge caused the container to become submerged. The rope was pulled out of the well and the container corked and labelled. The same technique was used to collect samples from the stream at various locations (at least 200 m apart and 3 m away from the bank). Prior to collection from a tap, the tap opening was cleansed with 70% alcohol. The tap was opened and water allowed to flow for a few seconds, to flush out any contaminants. Water samples were packed in an insulated box and transported to the laboratory.

Samples were convenience samples. The coordinates of sites were obtained using a Global Positioning System (Guangzhou Making Electronic Technology Co., Ltd, China) as this information was not available prior to our study. During subsequent sampling visits, a GPS was used to locate the sampling points. Wells investigated were not protected [16]. Tap water in study area is part of a large drinking water treatment system which receives adequate disinfection.

### Isolation and identification of isolates

*Vibrio cholerae* was isolated following enrichment in alkaline peptone water as described by Bag *et al*. [19] and Akoachere *et al*. [20]. Pure cultures were subjected to gram stain, motility, oxidase and catalase tests, and growth on Kliger Iron agar (KIA) slants. Their identity was confirmed using the Analytical Profile Index (API) 20E kits (BioMerieux SA, France). Isolates were serotyped using polyvalent *Vibrio cholerae* O1 and 139 antisera as described by CDC [21]. Isolates presenting a negative reaction with *V. cholerae* O1 polyvalent antiserum were typed using *V. cholerae* O139 polyavlent antiserum.

### Antibiotic Susceptibility Testing (AST)

The disc diffusion (Kirby-Bauer) technique was employed to test the susceptibility of isolates to antibiotics as previously described by the National Committee for Clinical Laboratory Standards (presently called Clinical and Laboratory Standards Institute) [22]. Antibiotics tested included: tetracyclines [tetracycline (30 μg), doxycycline (30 μg)], a β-lactam [amoxicillin (30 μg)], β-lactmase inhibitor [augmentin (30 μg)], folic acid synthesis inhibitor [trimethoprim-sulfamethoxazole (cotrimoxazole) (25 μg)], fluoroquinolones [ciprofloxacin (5 μg), ofloxacin (5 μg)] and protein synthesis inhibitor [chloramphenicol (30 μg)]. These antibiotics have been used for treatment during previous outbreaks of cholera in Douala [23, 24] or recommended for cholera treatment [25]. The diameters were compared with the recorded diameters of the control organism *E. coli* ATCC 25922 to determine susceptibility or resistance.

### Determination of physico-chemical characteristics of water samples

Physico-chemical parameters (temperature, pH and salinity) were determined on site immediately after sample collection. A thermometer (Chemistry Thermometer, No 3200, Germany) was inserted into the sample and temperature reading recorded after 3 minutes. pH was measured using a pH meter (HANNA Instrument, HI 9811, UK). The electrode of the instrument was rinsed with the water sample of interest, and then inserted into the sample and value recorded when the meter stabilized. Salinity values were obtained by measuring conductivity of samples (μS/cm), using a conductivity meter (HANNA Instrument, HI 9811, UK). Conductivity values were then converted to salinity (ppm).

### Data analysis

Statistical Package for Social Science (SPSS) version 15.0 was used to analyze data. The Chi-square (*X*^*2*^) test was employed to examine differences in the prevalence of *V. cholerae* in the water sources between seasons. The non-parametric analysis of variance (ANOVA) was used to describe and analyze variation in physico-chemical parameters. The Kendall *tau_b* non-parametric correlation was employed to study the strength and direction of association between physico-chemical parameters and occurrence of *V. cholerae*. Binary logistic regression model (forward stepwise method) was used to estimate the coefficient of predictors (physico-chemical characteristics) in association with *V. cholerae*. Differences were considered significant at P < 0.05.

## Results

### Contamination of water samples in the study area with *Vibrio cholerae*

Eighty-seven (27.4%) of the 318 samples analyzed were contaminated with *V. cholerae.* Stream samples (11/21, 52.4%) were more frequently contaminated than samples from other sources (Table 1). There was a significant difference in isolation of the organism from samples from various sources (*χ*^2^ = 8.830, df = 2, P = 0.012).

**Table 1:** **Contamination of water sources with** ***Vibrio cholerae***

Source	No. of samples analyzed	No. of samples positive (%)
Well (n = 45)	241	72 (29.9)
Tap (n = 8)	56	4 (7.1)
Stream (n = 1)	21	11 (52.4)
**Total**	**318**	**87 (27.4)**

Among the 96 isolates characterized as *V. cholerae,* 32 (33.3%) were *Vibrio cholerae* O1 while the rest 64 (66.7%) belonged to the non-O1/non-O139 serogroup (Table 2). *Vibrio cholerae* O1 was isolated only from stream and well water samples. The majority of isolates from stream were *V. cholerae* O1 (9/14, 64.3%). Isolates from tap water were serogroup non-O1/non-O139. Serogroup O139 was not isolated in our study. Among the 87 positive samples7 (8.1%) had O1 serogroup, 59 (67.8%) had non-O1 serogroup and 21 (24.1%) were contaminated with both serogroups. *Vibrio cholerae* was detected in 35 wells, 3 taps and the stream investigated. Wells W12, W13, W14, W27 and W37 (Figure 1) had the highest number of isolates with 5, 6, 7, 6 and 5 isolates respectively obtained from these sources (Additional file 1). Serogroup O1 was detected in 20 wells. *V. cholerae* O1 and non-O1/non-O139 co-existed in 13 (37.1%) wells and in the stream (Additional file 1).

**Table 2:** **Isolation of** ***V. cholerae*** **serogroups from water sources**

Source	No. of serogroup O1 isolates obtained	No. of serogroup non-O1/non-O139 isolates (%)	Total no. of ***V. cholerae***isolates (%)
Well	23 (29.5)	55 (70.5)	78 (81.3)
Tap	0 (0)	4 (100)	4 (4.2)
Stream	9 (64.3)	5 (35.5)	14 (14.6)
**Total**	**32 (33.3)**	**64 (66.7)**	**96 (100)**
**Statistics**	*χ* ^2^ = 16.237, df = 2; P = 0.000	*χ* ^2^ = 2.706; df = 2; P = 0.258	*χ* ^2^ = 8.830; df = 2; P = 0.012

### Seasonal distribution of *V. cholerae*in water sources

*V. cholerae* O1 and non-O1/non-O139 were detected in both the dry and rainy seasons in wells. Both serogroups occurred in the stream only in the rainy season (Table 3, Additional file 2). Overall, the frequency of isolation of the organism was significantly higher (*χ*^2^ = 7.009, df = 1, P = 0.008) in the rainy season (72/96, 75%) than in the dry season (24/96, 25%). There was a significant difference in isolation of the serogroups between seasons.

**Table 3:** **Seasonal distribution of** ***V. cholerae*** **isolates**

Season	Sample source	No. of isolates obtained (%)	Serogroup O1 (%)	Serogroup non-O1/non-O139
Dry season	Well	20 (20.8)	5 (5.2)	15 (15.6)
	Tap	1 (1)	0 (0)	1 (1)
	Stream	3 (3.1)	3 (3.1)	0 (0)
	**Total**	**24 (25)**	**8 (8.3)**	**16 (16.7)**
Rainy season	Well	58 (60.4)	18 (18.8)	40 (41.7)
	Tap	3(3.1)	0 (0)	3 (3.1)
	Stream	11 (11.5)	6 (6.3)	5 (5.2)
	**Total**	**72 (75)**	**24 (25)**	**48 (50)**

Overall isolation of *V. cholerae*: *χ* ^2^ = 7.009; df = 1; P = 0.008.

*V. cholerae* O1: *χ* ^2^ = 9.333, df = 1, P = 0.002.

*V. cholerae* non-O1/non-O139: *χ* ^2^ = 3.889, df = 1, P = 0.049.

### Physico-chemical characteristics of water samples and relationship with isolation of *Vibrio cholerae*

Mean temperature of samples in the dry season was 29.08°C, 27.73°C and 28.1°C for stream, tap and well respectively (Table 4). Generally temperature values dropped in the rainy season. Differences in temperature of samples from various sources were not significant (F = 2.301, P = 0.105). However, a significant difference was observed with season (F = 471.5, P<0.001). A significant positive correlation was observed between temperature and occurrence of *Vibrio cholerae* in the various water sources in the dry season (+0.248, P = 0.030). Correlation was positive in the rainy season (+0.012, P = 0.910) but weaker and not significant.

**Table 4:** **Physico-chemical characteristics of water samples**

Parameter	Season		Sample source			One-way ANOVA of means			
			Stream	Tap	Well	Sample source		Season	
						F	P	F	P
**Temperature (°C)**	**Dry**	**Mean ± SD**	29.08 ± 0.63	27.73 ± 0.42	28.15 ± 0.45	2.301	0.105	471.5	< 0.001
		**Range**	28.5-29.75	27.33-28.5	27.33-29.33				
	**Rainy**	**Mean ± SD**	26.17 ± 0.29	25.79 ± 0.21	26.41 ± 0.29				
		**Range**	26-26.5	25.5-26	25.67-27				
**pH**	**Dry**	**Mean ± SD**	6.87 ± 0.3	6.81 ± 0.15	5.96 ± 0.65	8.224	< 0.001	68.8	< 0.001
		**Range**	6.55-7.15	6.6-7.03	4.47-7.53				
	**Rainy**	**Mean ± SD**	7.58 ± 0.28	7.17 ± 0.04	6.91 ± 0.35				
		**Range**	7.3-7.85	7.13-7.23	6.03-7.87				
**Salinity(ppm)**	**Dry**	**Mean ± SD**	4.4 ± 0.45	1.6 ± 0.25	4.34 ± 2.23	22.49	< 0.001	5.63	0.019
		**Range**	3.96-4.85	1.47-2.22	1.42-14.99				
	**Rainy**	**Mean ± SD**	6.27 ± 0.57	1.79 ± 0.33	5.31 ± 1.43				
		**Range**	5.93-6.93	1.54-2.43	3.25-11.54				

Kendall’s tau_b:

Dry season: temperature and isolation of *V. cholerae*: +0.248, P = 0.030.

pH and isolation of *V. cholerae*: +0.184, P = 0.094.

Salinity and isolation of *V. cholerae*: +0.267, P = 0.015.

Rainy season: temperature and isolation of *V. cholerae*: +0.012, P = 0.910.

pH and isolation of *V. cholerae*: +0.090, P = 0. .379.

Salinity and isolation of *V. cholerae*: +0.223, P = 0.028.

Mean values of pH recorded in the dry season were 6.87, 6.81 and 5.96 for the stream, tap and well samples respectively (Table 4). Highest and lowest pH values in both seasons occurred in well water. Variation in pH of samples between seasons was significant (F = 68.8, P<0.001). Significant differences were also observed in the pH of samples from different sources (F = 8.224, P<0.001). pH positively correlated with isolation of the organism in the dry season (+0.184, P = 0.094) and in the rainy season (+0.09, P = 0.379) but this was not significant.

Mean values of salinity recorded in the dry season were 4.4, 1.6 and 4.34 ppm for the stream, tap and well samples respectively (Table 4). Salinity had a similar trend to pH with the highest and lowest values in the two seasons occurring in well water samples. There was a significant difference between salinity values obtained in the dry season and rainy season (F = 5.63, P = 0.019). Significant differences also occurred between samples from various sources (F = 22.49, P<0.001). A significant positive correlation between salinity and the isolation of *V. cholerae* was observed in the dry season (+0.267, P = 0.015) as well as in the rainy season (+0.223, P = 0.028).

In estimating the coefficient of physico-chemical characteristics of water samples and isolation of *V. cholerae* in a binary logistic regression using the forward-stepwise method with log likelihood function, it was observed that as pH (Wald = 11.753, df = 1, P = 0.001) increased, the odds of isolation of *V. cholerae* also increased (*B = 1.297, S.E = 0.378, Exp(B) = 3.657*) (Additional file 3). Salinity (score = 1.606, df = 1, P = 0.205) and temperature (score = 0.050, df = 1, P = 0.824) were insignificant to be included in the logistic regression equation (Additional file 3).

### Antibiotic susceptibility of *V. cholerae*isolates

All isolates (100%) were sensitive to the fluoroquinolones: ciprofloxacin and ofloxacin (Table 5). Chloramphenicol, doxycycline and augmentin showed good activities. Generally, O1 isolates had higher susceptibilities than non-O1/non-O139 (Table 4). Lower susceptibilities were recorded for tetracycline (62.5% and 50% for O1 and non-O1/non-O139 respectively), cotrimoxazole (65.6% and 42.2% respectively for O1 and non-O1/non-O139 *V. cholerae* isolates) and ampicillin (64% for non-O1/non-O139 serogroup).

**Table 5:** **Antibiotic susceptibility of** ***V. cholerae*** **isolates**

Antibiotic	Number resistant (%)		Number susceptible (%)	
	Serogroup O1	Serogroup non-O1/non-O139	Serogroup O1	Serogroup non-O1/non-O139
Amoxicillin	10 (31.3)	23 (36)	22 (68.8)	41 (64)
Augmentin	6 (18.8)	7 (11)	26 (81.3)	57 (89)
Ofloxacin	0 (0)	0 (0)	32 (100)	64 (100)
Ciprofloxacin	0 (0)	0 (0)	32 (100)	64 (100)
Tetracycline	12 (37.5)	32 (50)	20 (62.5)	32 (50)
Doxycycline	3 (9.4)	9 (14.1)	29 (90.6)	55 (85.9)
Cotrimoxazole	11 (34.4)	37 (57.8)	21 (65.6)	27 (42.2)
Chloramphenicol	2 (6.3)	2 (3)	30 (93.8)	62 (97)

Isolates showed multiple resistance (resistance to 2 or more drugs) to antibiotics. Four resistance patterns were observed among *V. cholerae* O1 isolates with patterns TE^R^ DXT^R^ (25%) and AML^R^ TE^R^ SXT^R^ (58.3%) being commonly encountered (Table 6). These patterns also predominated among the non-O1/non-O139 isolates. However, patterns AML^R^SXT^R^C^R^ and AML^R^AUG^R^C^R^ were found exclusively in *Vibrio cholerae* O1. Eleven (11) resistance patterns were unique to non-O1/non-O139 (Table 6).

**Table 6:** **Resistance patterns of** ***V. cholerae*** **isolates**

SEROGROUP O1		
**No.**	**Resistance pattern**	**No. exhibiting resistance pattern (%)**
R_1_^*^	TE ^R^ DXT ^R^	3 (25)
R_2_^*^	**AML** ^**R**^ **TE** ^**R**^ **SXT** ^**R**^	**7 (58.3)**
R_3_	AML ^R^ SXT ^R^ C ^R^	1 (8.3)
R_4_	AML ^R^ AUG ^R^ C^R^	1 (8.3)
**Total**		**12 (37.5)**
**SEROGROUP NON-O1/NON-O139**		
**No.**	**Resistance pattern**	**No. exhibiting resistance pattern (%)**
R_5_	AML ^R^ SXT^R^	4 (11.8)
R_6_	AML ^R^ TE ^R^	1 (2.9)
R_7_	AUG ^R^ TE ^R^	1 (2.9)
R_8_	AML ^R^ AUG ^R^	1 (2.9)
R_9_^*^	TE ^R^ DXT ^R^	4 (11.8)
R_10_	TE ^R^ SXT ^R^	4 (11.8)
R_11_	TE ^R^ DXT ^R^ SXT ^R^	2 (5.9)
R_12_^*^	**AML** ^**R**^ **TE** ^**R**^ **SXT** ^**R**^	**10 (29.4)**
R_13_	AML ^R^ AUG ^R^ SXT ^R^	1 (2.9)
R_14_	AUG ^R^ TE ^R^ SXT ^R^	2 (5.9)
R_15_	AML ^R^ TE ^R^ DXT ^R^ SXT ^R^	2 (5.9)
R_16_	AML ^R^ TE ^R^ SXT ^R^ C ^R^	1 (2.9)
R_17_	AML ^R^ TE ^R^ DXT ^R^ SXT ^R^ C ^R^	1 (2.9)
**Total**		**34 (53.1)**

AML, amoxicillin; AUG, augmentin; TE, tetracycline; DXT, doxycycline; SXT, trimethoprim/sulfamethoxazole; C, chloramphenicol; R, resistant; * resistance patterns detected in both *Vibrio cholerae* O1 and non-O1/non-O139 strains.

## Discussion

The city of Douala is well known as the main cholera-endemic area of Cameroon. It has experienced more than ten outbreaks of cholera since the arrival of the disease in Cameroon in 1971, most of which have originated from Bepanda [13, 14]. We analyzed water from various sources in Bepanda during a period with no known disease outbreak for the presence of *V. cholerae,* studied its susceptibility pattern to drugs used in cholera management and also determined the physico-chemical parameters associated with the presence of the organism in water in the study locality.

Eighty-seven samples (27.4%) were contaminated with *Vibrio cholerae. V. cholerae* is a normal inhabitant of natural aquatic environments such as rivers, estuaries and coastal waters [26] and has been detected in natural waters worldwide, including areas where clinical cases of cholera did not exist [27]. Samples from the stream were more contaminated. There was a significant difference in the prevalence of the organism in samples from various sources (Table 1). Most isolates were from wells. This is not surprising because recent reports [16] are showing deteriorating quality of water from dug-wells in study area. We could not access some wells and taps which were in premises with a gated fence since at times, there was no one within the premises to open the gate and let us in. The following wells were not sampled during the following months: March: W_13_, W_16_, W_25_, W_37_, W_40;_ April: W_1_, W_3_, W_9_, W_18_, W_21,_ W_45;_ May: W_10_, W_15_, W_24_, W_28_, W_33_, W_41,_ W_42;_ June: W_12_, W_7_, W_8_, W_14_, W_32;_ July: W_11_, W_22_, W_30_, W_39_, W_43_, W_44._ Initially we had 6 sampling points on the stream. After the first visit, access way to three of those points was blocked and we never sampled from there again. We also had more taps on the first visit but subsequently we were not able to sample from some of them.

Serological characterization of *V. cholerae* isolates identified 32 (33.3%) and 64 (66.7%) isolates belonging to serogroups O1 and non-O1/non-O139 respectively (Table 2). Both serogroups were isolated in the dry and rainy seasons (Table 3) indicating the presence of the organism all year round in study site. However, the majority of isolates were obtained during the rainy season. In Douala, the rainy season is characterized by torrential rains, which result in flooding and runoffs loaded with contaminants from diverse origins to water sources. The majority of the wells in Bepanda are poorly constructed and not well protected [16]. During periods of flood water may fill pit latrines and re-distribute faecal matter resulting in contamination of wells and surface water sources. Improvement of sanitation and access to safe water sources are urgently needed in Bepanda. In addition, continuous monitoring of water sources in study area is necessary for a better preparedness and control of cholera and cholera-like disease.

*V. cholerae* non-O1/non-O139 was isolated from all sources. Although non-O1/non-O139 strains do not cause epidemic cholera, they are recognized to be of public health relevance because they have been associated with sporadic cases or outbreaks of cholera-like diseases [3, 28] and many extra-intestinal infections [2, 29, 30]. The emergence of serogroup O139 as a second etiologic agent of cholera epidemics, along with the possible conversion of non-O1 to O1 serotype [31, 32] has increased interest in non-O1/non-O139 *V. cholerae* strains. Members of non-O1/non-O139 serogroup do not produce major virulence factors but genes coding for virulence factors have been identified in certain strains of both clinical and environmental origins [4, 5]. Thus, non-O1/non-O139 serogroup in the study area may constitute a public health risk. Studies are underway in our laboratory investigating the virulence potential of non-O1/non-O139 *Vibrio cholerae* from the aquatic environment of parts of the city of Douala where devastating outbreaks of cholera had occurred to show their possible involvement in diarrheal disease in these areas. All isolates from tap water were serogroup non-O1/non-O139. Municipal water distributed in Douala undergoes adequate treatment prior to distribution. Contamination of tap water could have been through a leakage along the distribution mains which we observed during sample collection. Thus prompt repairs of leaking pipes his highly solicited to prevent re-contamination of treated water.

Serogroup O139 was not detected in our study. Since the detection of this strain in 1992 as another cause of epidemic cholera [32], cholera caused by serogroup O139 had been limited to the Indian sub-continent and thus was believed to be of no public health importance in Africa. However, du Preez *et al*. [33] detected *V. cholerae* O139 strain in the estuarine environment of Mozambique indicating its spread out of the Indian sub-continent. These findings call for the inclusion of *V. cholerae* O139 in cholera surveillance in cholera endemic areas where this strain has never been reported.

Serogroup O1 was isolated only from wells and stream samples (Table 2). This is of great public health importance as toxigenic strains are responsible of cholera outbreaks and epidemics. Although we did not investigate the potential of our isolates to cause disease, their detection in waters is expected to serve as warning against an impending outbreak of cholera if appropriate preventive measures are not enforced. Inhabitants of the locality studied, therefore run the risk of experiencing cholera outbreak in the future, since most of them use well water for drinking, cooking and washing. There was no outbreak of cholera during the period of our study.

Environmental factors such as temperature, salinity, rainfall, sunlight, pH, concentration of ferric ions, exogenous products of algal growth and chitin have been associated with *V. cholerae* dynamics [34]. A significant positive correlation was observed between temperature and occurrence of *Vibrio cholerae* in the various water sources in the dry season (+0.248, P = 0.030) (Table 4). Correlation was positive in the rainy season (+0.012, P = 0.910) though weaker and not significant. pH positively correlated with isolation of the organism in the dry season (+0.184) and rainy season (+0.09) but correlation was not significant in both seasons (P = 0.094 and P = 0.379 for dry and rainy season respectively). Salinity had a significant positive correlation with the occurrence of the organism in the dry season (+0.267, P = 0.015) as well as rainy season (+0.223, P = 0.028) showing salinity to be strongly associated with *V. cholerae* isolation from water sources in Bepanda. However, salinity did not score significantly in the logistic regression model. Salinity has been demonstrated to influence significantly the growth of *V. cholerae* in cholera endemic areas [35, 36]. We did not explore lagged relationships eventhough previous studies have shown significant associations. pH (Wald = 11.753, df = 1, P = 0.001) was the only significant predictor from the binary logistic regression. Comprehensive studies would be necessary to evaluate the influence of pH on the presence of *V. cholerae* in the aquatic environment of Bepanda.

Isolates exhibited 100% susceptibility to fluoroquinolones: ciprofloxacin and ofloxacin. Augmentin, doxycycline and chloramphenicol also showed high activities in the two serogroups while cotrimoxazole, tetracycline and amoxicillin exhibited the lowest activities (Table 5). Resistance of clinical isolates from Douala to these antibiotics has been reported by Garrigue *et al*. [37]. Such resistant strains might have been disseminated into water sources. Studies in other parts of the world [38, 39] have reported varying susceptibilities of the organism to these antibiotics. Changes in susceptibility pattern have been attributed to isolation time, source and geographical location. However, high activities of ofloxacin and ciprofloxacin observed in our study are similar to other studies [15, 38, 39] and corroborate the use of fluoroquinolones as new alternatives for cholera treatment in some cholera endemic regions [40]. Our data and reports from other cholera endemic localities in Douala [20] therefore suggest ciprofloxacin and ofloxacin could be very good alternatives for chemotherapy during cholera or cholera-like disease outbreaks in Douala.

High resistance of O1 isolates to amoxicillin corroborates the report of Ngandjio *et al.* [20] on the emergence of resistance to amoxicillin toward the end of the 2005 cholera epidemic in Douala. Doxycycline and amoxicillin were extensively used during this outbreak as drugs of choice in chemotherapy and chemoprophylaxis [23, 24]. Prolonged usage of antibiotics for curative and prophylactic purposes during an epidemic can provoke the emergence and spread of resistant strains into the environment [41]. Overuse of antibiotics could also exert a selective pressure on pathogenic organisms and the bowel flora, which once shed, become an important reservoir of resistance genes that can be transferred to other bacteria such as *V. cholerae*. Genetic exchange has been reported to occur between *V. cholerae* and enterobacteriaceae in the environment such as *E. coli* that possess transposon and plasmid of the same group [42].

Four multi-drug resistance (MDR) patterns emerged among *V. cholerae* O1 isolates (Table 6). Isolates that were resistant to more than 3 drugs were not found. The patterns AML^R^ TE^R^ SXT^R^ and TE^R^ SXT^R^ were the most common resistance patterns in this serogroup. Akoachere *et al.* [20] recently detected multi-drug resistant toxigenic *V. cholerae* O1 in Douala. Ndayo *et al.* [15] reported the isolation of MDR O1 strains from water sources in Douala that exhibited resistance to up to five drugs: AML^R^ AUG^R^ TIC^R^ S^R^ C^R^ SXT^R^, a pattern similar to that obtained from clinical isolates during the 2004–2005 cholera epidemic. Like O1 isolates, multi-drug resistance was observed in the non-O1/non - O139 isolates. Thirteen MDR patterns were obtained (Table 6). Similar to serogroup O1, the pattern AML^R^ TE^R^ SXT^R^ predominated. We also observed that most non-O1/non-O139 isolates were simultaneously resistant to cotrimoxazole and tetracycline. The isolation of *V. cholerae* that were resistant to cotrimoxazole suggests the possible presence of the SXT element in the study area. The SXT element is a self-transmissible, chromosomally integrated genetic element which carries cross-resistance to cotrimoxazole, streptomycin and furazoloidine [43]. However, we did not test streptomycin and furazoloidine in our study. The SXT element has been demonstrated to acquire additional tetracycline and erythromycin resistance genes with high efficiency [44]. Thus, tetracycline and cotrimoxazole resistance observed in isolates could be due to the SXT element. From our results and other findings in Douala [15, 20] it would be necessary to investigate drug resistant *V. cholerae* from Douala for these resistance markers. However, the simultaneous resistance to tetracycline and cotrimoxazole could be rather related to the presence of both SXT element and resistance (R) plasmid known to carry tetracycline resistance genes [45]. The high prevalence of MDR *V. cholerae* in the water sources indicates the need for constant monitoring of the susceptibility pattern of the organism in cholera-endemic areas, in anticipation of an outbreak of cholera or gastroenteritis. It is necessary that preparedness activities for chemotherapy should include an efficient scheme for the management of antibiotics chosen, as this influences the emergence and prevalence of novel antibiotic resistant clones in cholera endemic areas.

### Limitations

We were not able to access all our sampling points during visits and this greatly affected our sample size. Analysis for toxin production or toxin production potential of isolates was not carried out. It is therefore difficult to associate these isolates with disease production particularly as they were all environmental isolates. We employed the disc diffusion technique to investigate the antibiotic susceptibility of isolates. MICs were not determined making it difficult to differentiate between strains that were very sensitive and those with higher MIC. This is important clinically because patients infected with strains whose MIC is high do not respond like those with very sensitive strains.

## Conclusions

Our study has shown that *V. cholerae* is present all year round in wells and streams in Bepanda. Since wells are a major source of water in study area and the stream is used for various activities which may expose residents to infection, cholera will continue to be a health threat in this locality if intervention measures to prevent outbreak are not implemented. Among the intervention measures, this locality should be considered a target for oral cholera vaccine as an immediate measure while resources are put together for WASH (water, sanitation and hygiene) improvement.

## Electronic supplementary material

Additional file 1: **Distribution of** ***V. cholerae*** **in contaminated water sources.** This shows the distribution of *Vibrio cholerae* isolated from various sources in study area. (XLSX 15 KB)

Additional file 2: **Monthly isolation of** ***Vibrio cholerae*** **from various sources.** This presents the monthly isolation of *V. cholerae* from each source. (XLSX 12 KB)

Additional file 3: Binary logistic regression analysis of physico-chemical parameters.(XLSX 10 KB)

Below are the links to the authors’ original submitted files for images.

Authors’ original file for figure 1

## Abbreviations

CDC: Centre for disease control and prevention
WASH: Water, sanitation and hygiene.
