# Supplementary figures and images for: Water sources as reservoirs of Vibrio choleraeO1 and non-O1 strains in Bepanda, Douala (Cameroon): relationship between isolation and physico-chemical factors
Source: BMC Infect Dis. 2014 Jul 30;14:421. doi: 10.1186/1471-2334-14-421 (PMC4131033; doi:10.1186/1471-2334-14-421)

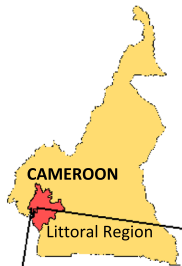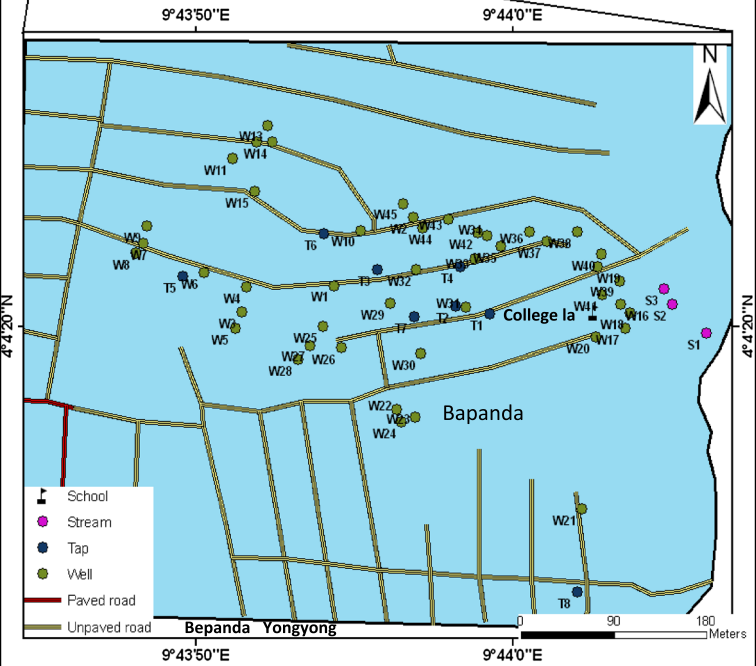

Supplement: Supplementary file 4 — Authors’ original file for figure 1 [file 12879_2013_3727_MOESM4_ESM.pdf]
